# Frailty Status, Sedentary Behaviors, and Risk of Incident Bone Fractures

**DOI:** 10.1093/gerona/glae186

**Published:** 2024-08-01

**Authors:** Jian Zhou, Rui Tang, Xuan Wang, Hao Ma, Xiang Li, Yoriko Heianza, Lu Qi

**Affiliations:** Department of Epidemiology, Tulane University School of Public Health and Tropical Medicine, New Orleans, Louisiana, USA; Department of Orthopedics, The Second Xiangya Hospital of Central South University, Changsha, China; Department of Epidemiology, Tulane University School of Public Health and Tropical Medicine, New Orleans, Louisiana, USA; Department of Epidemiology, Tulane University School of Public Health and Tropical Medicine, New Orleans, Louisiana, USA; Department of Epidemiology, Tulane University School of Public Health and Tropical Medicine, New Orleans, Louisiana, USA; Department of Epidemiology, Tulane University School of Public Health and Tropical Medicine, New Orleans, Louisiana, USA; Department of Epidemiology, Tulane University School of Public Health and Tropical Medicine, New Orleans, Louisiana, USA; Department of Epidemiology, Tulane University School of Public Health and Tropical Medicine, New Orleans, Louisiana, USA; Department of Nutrition, Harvard T.H. Chan School of Public Health, Boston, Massachusetts, USA

**Keywords:** Bone fractures, Physical frailty, Sedentary behavior time

## Abstract

**Background:**

The associations of physical pre-frailty and frailty with bone fractures and the modified effect of sedentary lifestyle remain uncertain. This study was performed to explore the association of physical pre-frailty and frailty with risk of incident bone fractures, and test the modification effects of sedentary lifestyle and other risk factors.

**Methods:**

This cohort study included 413 630 participants without bone fractures at baseline in the UK Biobank study between 2006 and 2010 and followed up to 2021. The mean age of the participants was 56.5 years. A total of 224 351 (54.2%) enrolled participants were female and 376 053 (90.9%) included participants were White. Three Cox regression models were constructed to analyze the association of pre-frailty and frailty with total fractures, hip fractures, vertebrae fractures, and other fractures.

**Results:**

As compared with the physical nonfrailty group, the multivariate-adjusted hazard ratios were 1.17 (95% confidence interval [CI]: 1.14–1.21) and 1.63 (95% CI: 1.53–1.74) for the physical pre-frailty group and frailty group, respectively (*p-*trend < .001). In addition, we found that sedentary behavior time significantly accentuated the associations of physical pre-frailty and frailty with total fractures (*p-*interaction <.001), hip fractures (*p-*interaction = .013), and other fractures (*p-*interaction <.001).

**Conclusions:**

Our results indicate that physical pre-frailty and frailty are related to higher risks of bone fractures; such association was more pronounced among those with longer sedentary behavior time.

Bone fractures pose a significant public health concern and are the leading cause of disability in older adults. Among individuals aged over 50 years, it is estimated that 1 in 3 women and 1 in 5 men suffer from osteoporotic fractures (1). Bone fractures affect multiple sites including the hip, wrist, arm, spine, leg, and ankle, with the most common hip fractures accounting for 18.2% (1.63 million) of all fractures (2). The incidence cases of hip fractures worldwide are projected to nearly double by 2050, compared to 2018 (3). To identify modifiable risk factors for fractures is crucial for lowering the incidence of the disorders.

Physical frailty is a condition characterized by the presence of 5 indicators: weight loss, exhaustion low physical activity, slow walking pace, and low grip strength (4). Individuals with 1 or 2 indicators are classified as physical pre-frailty (5–7). Emerging evidence has associated physical frailty with higher risks of fall and osteoporosis (8–10). However, limited studies tested the relationship of physical frailty with bone fractures in multiple sites and few studies analyzed the association between physical pre-frailty and bone fractures. Additionally, no study has investigated the potential interactions between physical frailty and sedentary lifestyle in relation to bone fractures.

Sedentary lifestyle including prolonged sitting or lying down has been related to both decreased bone mineral density (BMD) (11) and high risks of pre-frailty and frailty (12). We hypothesized that sedentary lifestyle might accentuate the relationship of physical pre-frailty and frailty with risk of bone fractures. To date, no study has assessed the interaction between sedentary behavior time and physical pre-frailty/frailty status in relation to risk of incident bone fractures.

In this study, we analyzed the associations of physical pre-frailty and frailty with bone fractures at multiple sites (total fractures, hip fractures, vertebrae fractures, and other fractures) among 413 630 middle- and old-aged adults without bone fractures at baseline. We particularly tested the interaction of physical frailty status with sedentary behavior time and other risk factors in relation to risk of incident bone fractures.

## Method

### Study Population

The United Kingdom Biobank (UK Biobank) is a large-scale biomedical cohort research database initiated and established by the U.K. government. The database includes over 500 000 participants aged 40–69 who were recruited across the United Kingdom from 2006 to 2010. The UK Biobank includes genetic, living environment, and health data based on a large sample population through the collection of participants’ biological samples and questionnaires. The UK Biobank is accessible to authorized researchers and scientists around the world for the purpose of performing research focused on chronic diseases that seriously threaten human health (13). This study was approved by the Institutional Review Board of a major university.

### Assessment of Frailty

We evaluated the frailty index using 5 frailty indicators including weight loss, exhaustion, physical activity, walking pace, and grip strength. (1) Grip strength was measured using a Jamar J00105 hydraulic hand dynamometer, and the lower value from the 2 hands’ measurements was used. Low grip strength was defined based on sex- and body mass index (BMI)-adjusted cutoffs. The other 4 variables included in the frailty phenotype were defined through an online touchscreen questionnaire. (2) For weight loss, participants were asked, “Compared with one year ago, has your weight changed?” Those who responded with “Yes, lost weight” were classified as having weight loss, whereas responses of “No, weigh about the same” and “Yes, gained weight” were classified as not having weight loss. (3) For exhaustion, participants were asked, “Over the past two weeks, how often have you felt tired or had little energy?” Those who selected “More than half the days” or “Nearly every day” were classified as experiencing exhaustion, whereas those who chose “Not at all” or “Several days” were not. (4) For walking pace, the question was, “How would you describe your usual walking pace?” Participants who answered “Slow pace” instead of “Steady average pace” or “Brisk pace” were classified as having a slow walking pace. (5) Physical activity was measured with the question, “In the last 4 weeks, did you spend any time doing the following?” Responses were categorized into none (no physical activity), low (light DIY activity, such as pruning or watering the lawn), medium (heavy DIY activity like weeding, lawn mowing, carpentry, and digging; walking for pleasure or other exercises like swimming, cycling, fitness routines, or bowling), and high (strenuous sports). Another question, “How many times in the last 4 weeks did you do light DIY?” was asked. Participants who reported none or light activity with a frequency of once per week or less were considered to have low physical activity.

The frailty index ranges from 0 to 5, and detailed information on frailty index criteria is indicated in Supplementary Table 1. Participants who did not meet any of the criteria were designated as nonfrail (frailty index = 0). The participants who met 1 or 2 criteria were classified as pre-frail (frailty index = 1 or 2) and those who met 3–5 criteria were categorized as frail (frailty index = 3 to 5).

### Participant Selection

In the present study, 502 411 participants were recruited from the UK Biobank. Thirty-two participants withdrew from UK Biobank and 34 526 participants without data for frailty were removed. After excluding these participants, 11 639 participants who were diagnosed with bone fractures at baseline were removed from the cohort. Then 42 584 participants with self-reported bone fractures were deleted. Finally, a total of 413 630 participants were included in our study (Supplementary Figure 1).

### Evaluation of Other Variables

The UK Biobank used Assessment Centre Environment touch questionnaires to obtain the basic characteristics of participants: age, sex, and ethnic background, Townsend Deprivation Index, household income, smoking status, alcohol intake, falls history, vitamin D supplementation, calcium supplementation. BMI was calculated as the weight in kilograms divided by height in meters squared, and standing height was measured using a Seca 202 device. The T-score was derived from the measurement of BMD in the heel using ultrasound, and it indicated an individual’s bone density compared to the expected level for someone of the same gender. The T-score was expressed in standard deviations (*SD*) from the standard, providing a numerical representation of the deviation of the individual’s bone density from the norm. Serum vitamin D was measured by chemiluminescent immunoassay (CLIA) analysis on a DiaSorin Ltd. LIASON XL and serum calcium were measured by Arsenazo III analysis on a Beckman Coulter AU5800.

The frequency categories for meat and fish were recoded as follows: “never” as 0, “less than once a week” as 0.5, “once a week” as 1, “2–4 times a week” as 3, “5–6 times a week” as 5.5, and “once or more daily” as 7. The frequency of consumption of unprocessed red meat was calculated by summing the servings for beef, lamb/mutton, and pork. For vegetables and fruits, participants were asked about the amount they consumed in heaped tablespoons or pieces per day, which were then totaled for cooked/salad vegetables, raw vegetables, fresh fruit, and dried fruit. Healthy diet score was determined based on vegetable intake of at least 4 tablespoons per day, fruit intake of at least 3 pieces per day, fish intake of at least twice a week, unprocessed red meat intake of no more than twice a week, and processed meat intake of no more than twice a week. Each favorable dietary factor was given 1 point, and the total score ranged from 0 to 5, which has been described in our previous study.

For sedentary behavior time, participants were asked to use a touchscreen questionnaire to answer these 3 inquiries regarding their sedentary behavior: On an average day, how long do you engage in watching TV, using a computer (nonwork related), or driving? The sum of time spent on these 3 activities was calculated to determine the total sedentary behavior time. Based on the total time, we divided the sedentary behavior into 3 categories: less than 3 h/d, 3–5 h/d, and ≥6 h/d. Details of these evaluations are accessible at the UK Biobank website (www.ukbiobank.ac.uk).

### Assessment of Outcomes

The outcome assessed in this study was total fractures and the secondary outcomes analyzed were hip fractures, vertebrae fractures, and other fractures. Total fractures is the sum of the hip, vertebrae, and other fractures. We identified incident total fractures by referring to hospital admission records and using the International Classification of Diseases, 10th revision (ICD-10) codes, as outlined in Supplementary Table 2. We only used ICD-9 codes and self-reported diagnoses to confirm the presence of bone fractures at baseline, for the purpose of excluding them from the incident analyses (Supplementary Table 3). Bone fractures that occurred at the skull, face, hands, and feet and were typically caused by trauma, as well as by malignancy, atypical femoral fractures, periprosthetic fractures, and previously healed fractures, were excluded from the current analysis (14). However, we did not exclude traumatic fractures because the reasons for the trauma were not well documented in the ICD-10 codes.

We determined the date of bone fractures by analyzing data from the cumulative medical records of hospital diagnoses. The UK Biobank constructed a comprehensive linkage for the data of mortality status. Furthermore, the information on reasons and dates for hospitalization can be used through the linkage to Scottish morbidity records for Scottish participants and health event statistics for England and Wales participants. More information is accessible at https://digital.nhs.uk/services. All participants enrolled were followed up from the date of recruitment (between 2006 and 2010) to the earliest occurrence of diagnosis of bone fractures (up to November 27, 2021), loss to follow-up, or death.

### Statistical Analysis

Continuous variables were expressed as the mean ± *SD*, and all categorical variables were expressed as the count with percentage. Three Cox regression models were constructed to analyze the association of frailty status with total fractures, hip fractures, vertebrae fractures, and other fractures. Model 1 was adjusted for baseline age (years) and sex (male or female). Based on Model 1, Model 2 was further adjusted for ethnic background (White or others), Townsend Deprivation Index (continuous), household income (<£18 000, £18 000–£30 999, £31 000–£51 999, £52 000–£100 000, or >£100 000), BMI (continuous), standing height (continuous), smoking status (never, previous, or current smoking), alcohol intake (<1, 1–2, >2 times/week), healthy diet score (<3 or ≥3), and sedentary behavior time (continuous). Based on Model 2, Model 3 was further adjusted for heel BMD T-score (continuous), falls history (with or without), vitamin D supplementation (yes or no), calcium supplementation (yes or no), serum vitamin D (continuous), and serum calcium (continuous). For categorical variables, the missing data of covariates were coded as a missing indicator category, and mean values were adopted for missing data of continuous variables. Supplementary Table 4 indicated the numbers and percentages of participants with missing covariates.

Then, we performed a series of subgroup analyses stratified by age (≥60 vs <60 years), sex (male vs female), ethnic background (White vs others), Townsend Deprivation Index (≥median vs <median), household income (≥31 000 vs <31 000), BMI (18.5–24.9, 25–29.9, vs ≥30 kg/m^2^), standing height (≥median vs <median), smoking status (never, previous, vs current), alcohol intake (<1, 1–2, vs >2 times/week), healthy diet score (≥3 vs <3), sedentary behavior time (<3, 3–5, vs ≥6 hours), heel BMD T-score (<−2.5, −2.5 to 1 vs >−1 times/week), and fall history (with vs without). We used the same Cox model by adding interaction terms.

### Sensitivity Analysis

We conducted 3 sensitivity analyses to explore the robustness of our study. First, we deleted the participants who reported bone fractures in the first 2 years of the follow-up. Second, we excluded the participants with missing covariate data. Third, all the missing data were imputed using multiple imputation with chained equations. All results were expressed as the HR and 95% CI. SAS version 9.4 (SAS Institute, Cary, NC) was used to perform the statistical analysis, and we considered a 2-sided *P* value of <.05 as indicating statistically significant differences.

## Results

### Baseline Characteristics

Table 1 shows the baseline characteristics of the included participants. The mean age of the participants was 56.5 years and a total of 224 351 (54.2%) enrolled participants were female. 243 798 (58.9%), 155 470 (37.6%), and 14 362 (3.5%) participants were physical nonrailty, pre-frailty, and frailty, respectively. Frail participants were more likely to be women, non-White, with low household income, high BMI, low standing height, current smokers, with low healthy diet scores, with long sedentary behavior time, with low heel BMD T-scores, and users of vitamin D and calcium.

**Table 1.** Baseline Features of Participants

Characteristics	Total (*n* = 413 630)	Physical frailty status		
		Nonfrailty (*n* = 243 798)	Pre-frailty (*n* = 155 470)	Frailty (*n* = 14 362)
Physical frailty indicators, *n* (%)				
Weight loss	63 147 (15.3)	0 (0.0)	56 901 (36.6)	6 246 (43.5)
Exhaustion	50 773 (12.3)	0 (0.0)	40 772 (26.2)	10 001 (69.6)
Low physical activity	36 166 (8.7)	0 (0.0)	26 509 (17.1)	9 657 (67.2)
Slow walking pace	31 019 (7.5)	0 (0.0)	19 951 (12.8)	11 068 (77.1)
Low grip strength	57 069 (13.8)	0 (0.0)	47 002 (30.2)	10 067 (70.1)
Age, years, mean (*SD*)	56.5 (8.1)	56.2 (8.1)	56.7 (8.1)	57.9 (7.6)
Female, *n* (%)	224 351 (54.2)	126 818 (52.0)	88 548 (57.0)	8 985 (62.6)
Ethnic background, *n* (%)				
Other	36 284 (8.8)	18 621 (7.6)	15 730 (10.1)	1 933 (13.5)
White	376 053 (90.9)	224 499 (92.1)	139 205 (89.5)	12 349 (86.0)
Townsend Deprivation Index, mean (*SD*)	−1.4 (3.0)	−1.7 (2.8)	−1.0 (3.2)	0.5 (3.6)
Household income, £, *n* (%)				
<18 000	77 552 (18.8)	36 358 (14.9)	35 201 (22.6)	5 993 (41.7)
18 000–30 999	90 834 (22.0)	52 704 (21.6)	35 465 (22.8)	2 665 (18.6)
31 000–51 999	94 858 (22.9)	60 132 (24.7)	33122 (21.3)	1 604 (11.2)
52 000–100 000	74 549 (18.0)	50 807 (20.8)	22 997 (14.8)	745 (5.2)
>100 000	19 802 (4.8)	14 219 (5.8)	5 457 (3.5)	126 (0.9)
Body mass index, kg/m^2^, mean (*SD*)	27.4 (4.8)	26.6 (4.1)	28.3 (5.1)	31.3 (6.7)
Stand height, cm, mean (*SD*)	168.5 (9.3)	169.5 (9.1)	167.4 (9.3)	164.4 (9.1)
Smoking status, n (%)				
Never	227 164 (54.9)	138 208 (56.7)	82 392 (53.0)	6 564 (45.7)
Previous	143 637 (34.7)	84 046 (34.5)	54 568 (35.1)	5 023 (35.0)
Current	41 548 (10.0)	20 946 (8.6)	17 932 (11.5)	2 670 (18.6)
Alcohol intake, times/week, *n* (%)				
<1	124 478 (30.1)	59 728 (24.5)	56 367 (36.3)	8 383 (58.4)
1–2	107 288 (25.9)	63 867 (26.2)	40 471 (26.0)	2 950 (20.5)
>2	181 629 (43.9)	120 134 (49.3)	58 499 (37.6)	2 996 (20.9)
Healthy diet score, *n* (%)				
<3	131 962 (31.9)	75 685 (31.0)	50 832 (32.7)	5 445 (37.9)
≥3	267 719 (64.7)	162 016 (66.5)	98 006 (63.0)	7 697 (53.6)
Sedentary behavior time, hours, mean (*SD*)	4.8 (2.4)	4.6 (2.2)	5.1 (2.5)	5.8 (3.1)
Heel BMD T-score, mean (*SD*)	−0.3 (1.3)	−0.3 (1.2)	−0.3 (1.3)	−0.4 (1.4)
With history of falls, *n* (%)	72 916 (17.6)	35 112 (14.4)	31 896 (20.5)	5 908 (41.1)
Vitamin D, *n* (%)	15 770 (3.8)	8 856 (3.6)	6 220 (4.0)	694 (4.8)
Calcium supplementation, *n* (%)	26 886 (6.5)	15 115 (6.2)	10 580 (6.8)	1 191 (8.3)
Serum vitamin D, mmol/L mean (*SD*)	48.8 (21.0)	50.4 (20.8)	47.1 (21.0)	39.8 (20.7)
Serum calcium, mmol/L, mean (*SD*)	2.4 (0.1)	2.4 (0.1)	2.4 (0.1)	2.4 (0.1)

*Notes*: BMD = bone mineral density; SD = standard deviation

### Relationship of Physical Frailty Status With Bone Fractures

The median follow-up time was 12.7 years. A total of 19 983 bone fractures were reported, including 3 378 hip fractures, 1 843 vertebrae fractures, and 16 299 other fractures. Supplementary Figure 2 shows the cumulative hazard curves for the probability of bone fractures among physical nonfrailty, pre-frailty, and frailty participants. After adjusting for age, sex, ethnic background, Townsend Deprivation Index, household income, BMI, standing height, smoking status, alcohol intake, healthy diet score, sedentary behavior time, heel BMD T-score, falls history, vitamin D supplementation, calcium supplementation, serum vitamin D, and serum calcium, the results from Model 3 indicated that the adjusted hazard ratio (HR) related to total fractures was 1.17 (95% confidence interval [CI]: 1.14–1.21) for pre-frailty and 1.63 (95% CI: 1.53–1.74) for frailty, respectively. Both physical pre-frailty and frailty were related to an increased risk of total fractures, hip fractures, vertebrae fractures, and other fractures (Table 2).

**Table 2.** Hazard Ratios and 95% Confidence Intervals for Association of Physical Pre-frailty and Frailty With Outcome of Fractures

Outcomes	Physical Frailty Status			*p*-Trend
	Nonfrailty	Pre-frailty	Frailty	
Total fractures				
Event, *n* (%)	10 585 (4.3)	8 220 (5.3)	1 178 (8.2)	
Model 1	1 (Reference)	1.19 (1.16–-1.23)	1.84 (1.73–-1.95)	<.001
Model 2	1 (Reference)	1.21 (1.17–1.24)	1.83 (1.72–1.95)	<.001
Model 3	1 (Reference)	1.17 (1.14–1.21)	1.63 (1.53–1.74)	<.001
Hip fractures				
Event, *n* (%)	1 704 (0.7)	1 440 (0.9)	234 (1.6)	
Model 1	1 (Reference)	1.26 (1.18–1.35)	2.13 (1.86–2.44)	<.001
Model 2	1 (Reference)	1.35 (1.26–1.45)	2.39 (2.07–2.77)	<.001
Model 3	1 (Reference)	1.30 (1.21–1.39)	2.03 (1.76–2.35)	<.001
Vertebrae fractures				
Event, *n* (%)	849 (0.4)	828 (0.5)	166 (1.2)	
Model 1	1 (Reference)	1.53 (1.39–1.68)	3.35 (2.84–3.96)	<.001
Model 2	1 (Reference)	1.46 (1.32–1.62)	2.85 (2.38–3.41)	<.001
Model 3	1 (Reference)	1.42 (1.28–1.57)	2.51 (2.09–3.01)	<.001
Other fractures				
Event, *n* (%)	8 750 (3.6)	6 653 (4.3)	896 (6.2)	
Model 1	1 (Reference)	1.17 (1.13–1.21)	1.69 (1.58–1.81)	<.001
Model 2	1 (Reference)	1.17 (1.13–1.21)	1.66 (1.55–1.79)	<.001
Model 3	1 (Reference)	1.14 (1.10–1.18)	1.48 (1.38–1.60)	<.001

*Notes*: Model 1: adjusted for age (years) and sex (male or female). Model 2: Model 1 + ethnic background (White or others), Townsend Deprivation Index (continuous), household income (<£18 000, £18 000–£30 999, £31 000–£51 999, £52 000–£100,000, or >£100 000), body mass index (continuous), standing height (continuous), smoking status (never, previous or current smoking), alcohol intake (<1, 1–2, >2 times/week), healthy diet score (<3 or ≥3), and sedentary behavior time (continuous). model 3: Model 2 + heel bone mineral density T-score (continuous), falls history (with or without), vitamin D supplementation (yes or no), calcium supplementation (yes or no), serum vitamin D (continuous), and serum calcium (continuous).

**Figure 1.**
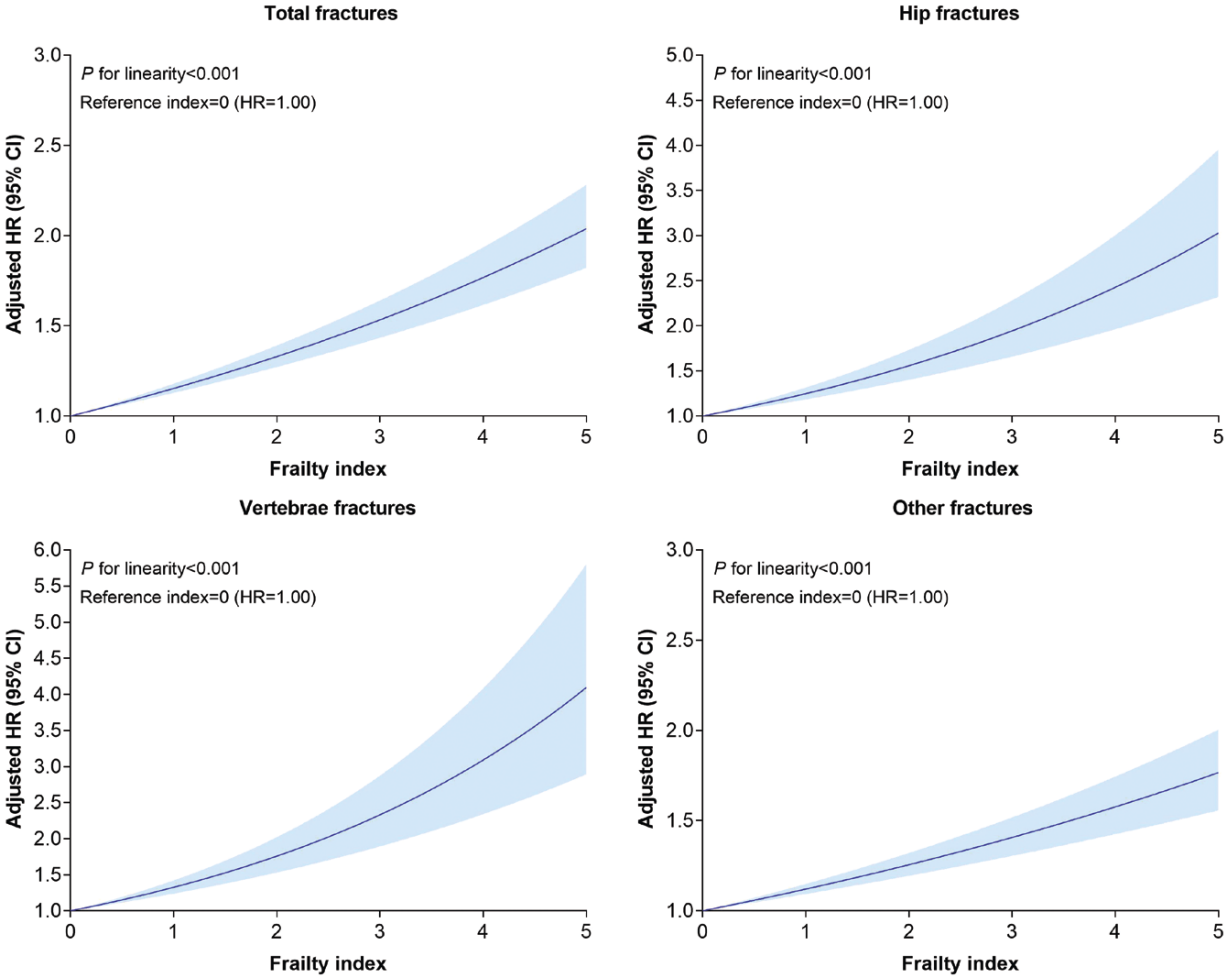
Dose–response associations of physical frailty index with risk of bone fractures. Model 3 with penalized splines adjusted for age (years), sex (male or female), ethnic background (White or others), Townsend deprivation index (continuous), household income (<£18 000, £18 000–£30 999, £31 000–£51 999, £52 000–£100 000, or >£100 000), body mass index (continuous), standing height (continuous), smoking status (never, previous or current smoking), alcohol intake (<1, 1–2, >2 times/week), healthy diet score (<3 or ≥3), sedentary behavior time (continuous), heel bone mineral density T-score (continuous), falls history (with or without), vitamin D supplementation (yes or no), calcium supplementation (yes or no), serum vitamin D (continuous), and serum calcium (continuous).

In restricted cubic splines, we observed a positive linear relationship between physical frailty index and incidence of total fractures (*p*-linearity < .001), hip fractures (*p*-linearity < .001), vertebrae fractures (*p*-linearity < .001), and other fractures (*p*-linearity < 0.001; Figure 1). Furthermore, the association of individual physical frailty indicators with risk of bone fractures was analyzed and slow walking pace showed the strongest association for bone fractures (Supplementary Figure 3).

### Effect Modifications by Sedentary Lifestyle and Other Risk Factors

**Figure 2.**
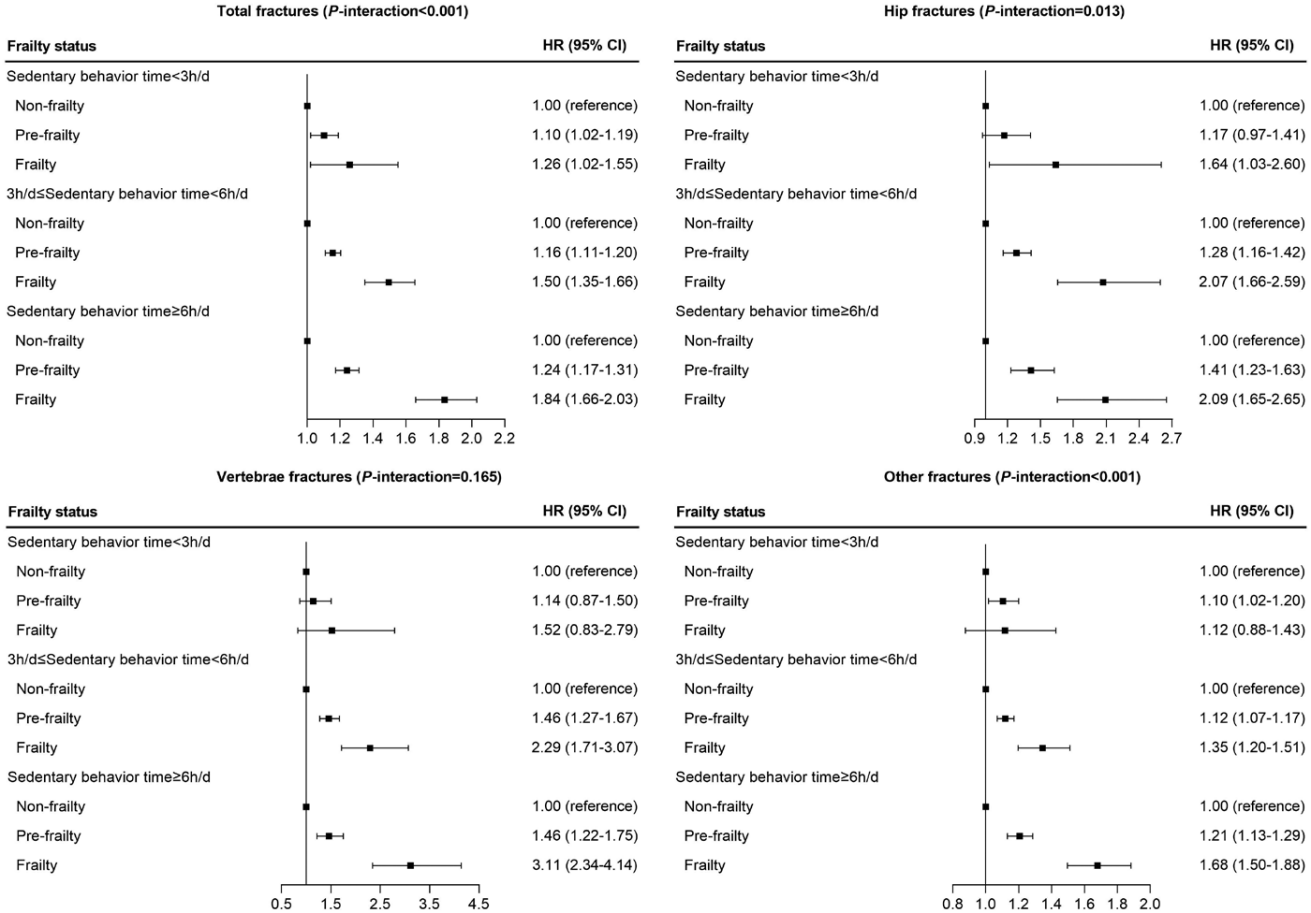
Association of physical frailty status with risk of fractures stratified by sedentary behavior time via Model 3 adjusted for age (years), sex (male or female), ethnic background (White or others), Townsend deprivation index (continuous), household income (<£18 000, £18 000–£30 999, £31 000–£51 999, £52 000–£100 000, or >£100 000), body mass index (continuous), standing height (continuous), smoking status (never, previous or current smoking), alcohol intake (<1, 1–2, >2 times/week), healthy diet score (<3 or ≥3), sedentary behavior time (continuous), heel bone mineral density T-score (continuous), falls history (with or without), vitamin D supplementation (yes or no), calcium supplementation (yes or no), serum vitamin D (continuous), and serum calcium (continuous).

We tested interactions of physical frailty status with sedentary behavior time in relation to risk of incident total fractures, hip fractures, vertebrae fractures, and other fractures. The results indicated that the associations of physical pre-frailty and frailty with total fractures, hip fractures, and other fractures were significantly accentuated by sedentary behavior time (*p*-interaction < .05; Figure 2). The association of physical pre-frailty and frailty with total fractures was stronger among participants with sedentary behavior time of more than 6 h/d than other groups with less sedentary behavior time. Among the individual components defining physical frailty indicators, weight loss, exhaustion, and low grip strength showed similar interaction patterns with sedentary behavior time on risk of total fractures (Supplementary Figure 4).

Moreover, in the joint analysis of frailty status and sedentary behavior time with risk of incident bone fractures (Supplementary Figure 5), we observed that participants with frailty and sedentary behavior time >6 h/d had the highest risk of incident bone fractures, with an HR of 1.81 (95% CI: 1.60–2.04) for total fractures, 2.70 (95% CI: 2.02–3.61) for hip fractures, 2.31 (95% CI: 1.61–3.31) for vertebrae fractures, and 1.63 (95% CI: 1.43–1.87) for other fractures.

Additionally, the results from stratified analyses demonstrated that the association of physical pre-frailty and frailty with total fractures was strongest among participants who were older than 60 years (*p-*interaction = .014), men (*p-*interaction < .001), with low household income (*p-*interaction < .001), and current smoker (*p-*interaction = .001; Table 3).

**Table 3.** Association of Physical Pre-frailty and Frailty With Risk of Bone Fractures by Potential Risk Factors Via Model 3^*^.

Subgroup	Physical Frailty Status			*p*-Trend	*p-*Interaction
	Nonfrailty	Pre-frailty	Frailty		
Age (years)					.014
<60	1 (references)	1.14 (1.09–1.19)	1.67 (1.50–1.85)	<.001	
≥60	1 (references)	1.20 (1.16–1.25)	1.65 (1.52–1.80)	<.001	
Sex					<.001
Women	1 (references)	1.15 (1.11–1.19)	1.55 (1.43–1.68)	<.001	
Men	1 (references)	1.20 (1.14–1.26)	1.79 (1.60–2.01)	<.001	
Townsend deprivation index					.967
<Median	1 (references)	1.20 (1.15–1.25)	1.68 (1.50–1.89)	<.001	
≥Median	1 (references)	1.15 (1.10–1.20)	1.61 (1.49–1.74)	<.001	
Household income (£)					<.001
<31 000	1 (references)	1.18 (1.13–1.23)	1.71 (1.58–1.86)	<.001	
≥31 000	1 (references)	1.12 (1.07–1.18)	1.50 (1.26–1.79)	<.001	
Body mass index (kg/m^2^)					.467
18.5–24.9	1 (references)	1.17 (1.11–1.23)	1.64 (1.42–1.89)	<.001	
25–29.9	1 (references)	1.16 (1.10–1.21)	1.65 (1.48–1.84)	<.001	
≥30	1 (references)	1.20 (1.13–1.28)	1.61 (1.45–1.78)	<.001	
Standing height^†^					.103
<Median	1 (references)	1.15 (1.10–1.21)	1.68 (1.54–1.82)	<.001	
≥Median	1 (references)	1.18 (1.13–1.23)	1.50 (1.35–1.66)	<.001	
Smoking status					.001
Never	1 (references)	1.17 (1.12–1.22)	1.49 (1.35–1.65)	<.001	
Previous	1 (references)	1.14 (1.09–1.20)	1.67 (1.50–1.85)	<.001	
Current	1 (references)	1.29 (1.18–1.41)	1.89 (1.63–2.20)	<.001	
Alcohol intake (times/week)					.073
<1	1 (references)	1.20 (1.14–1.27)	1.62 (1.48–1.78)	<.001	
1–2	1 (references)	1.14 (1.08–1.21)	1.63 (1.41–1.87)	<.001	
>2	1 (references)	1.16 (1.11–1.22)	1.73 (1.53–1.96)	<.001	
Healthy diet score					.055
<3	1 (references)	1.13 (1.07–1.20)	1.63 (1.46–1.81)	<.001	
≥3	1 (references)	1.17 (1.13–1.22)	1.59 (1.46–1.74)	<.001	
Heel BMD T-score					.387
>−1	1 (references)	1.15 (1.11–1.21)	1.71 (1.56–1.88)	<.001	
−2.5 to 1	1 (references)	1.20 (1.14–1.26)	1.53 (1.37–1.71)	<.001	
<−2.5	1 (references)	1.23 (1.04–1.47)	1.98 (1.50–2.60)	<.001	
Fall history					.172
Without	1 (references)	1.14 (1.10–1.18)	1.62 (1.49–1.77)	<.001	
With	1 (references)	1.26 (1.19–1.34)	1.66 (1.50–1.84)	<.001	

^*^Model 3: adjusted for age (years), sex (male or female), ethnic background (white or others), Townsend deprivation index (continuous), household income (<£18 000, £18 000–£30 999, £31 000–£51 999, £52 000–£100 000, or >£100 000), body mass index (continuous), standing height (continuous), smoking status (never, previous or current smoking), alcohol intake (<1, 1–2, >2 times/week), healthy diet score (<3 or ≥3), sedentary behavior time (continuous), heel bone mineral density T-score (continuous), falls history (with or without), vitamin D supplementation (yes or no), calcium supplementation (yes or no), serum vitamin D (continuous) and serum calcium (continuous).

^†^The median value of standing height was calculated separately by sex.

### Sensitivity Analyses

Several sensitivity analyses were conducted to show the robustness of results from the multivariable model. The results remained stable after excluding participants with incidence of bone fractures during the first 2 years of follow-up (Supplementary Table 5). When we removed the participants with missing covariates, the results from the Cox proportional hazard models did not change significantly (Supplementary Table 6). Additionally, we observed that the results did not change obviously after including imputed data for all missing covariate data using multiple imputation (Supplementary Table 7).

## Discussion

In this prospective study of a total of 413 630 middle- and old-aged adults, we found that physical pre-frailty and frailty were related to a 17% and a 63% higher risk of total fractures, respectively, as compared with physical nonfrailty. We found that both physical pre-frailty and frailty were significantly related to higher risks of site-specific fractures including hip fractures, vertebrae fractures, and other fractures compared to physical nonfrailty. In addition, we found that the associations of physical pre-frailty and frailty with total fractures, hip fractures, and other fractures were significantly accentuated by sedentary behavior time, and other risk factors including household income and smoking.

In line with our findings, several previous studies found that physical frailty status was related to an increased risk of bone fractures including hip fractures (15,16). However, few studies explored the association of physical frailty with bone fractures at multiple sites and limited studies analyzed the association between physical pre-frailty and bone fractures. In the current study, our findings expand this knowledge by showing both physical pre-frailty and frailty were associated with increased risks of total fractures and fractures at multiple sites including hip fractures, vertebrae fractures, and other fractures.

Although the precise mechanisms underlying the positive association between frailty and incident bone fractures have not yet been fully understood, several possible reasons may explain such association. Frail people are more likely to experience falls and subsequent hip and vertebrae fractures due to their decreased physical function and increased risk of mobility impairments (16). In addition, the decreased physical function associated with physical frailty may contribute to postural instability and poor balance, further increasing the risk of falls, and leading to hip and vertebrae fractures, particularly in those with low bone density or osteoporosis (16). Moreover, hip fractures may further exacerbate physical frailty by leading to prolonged hospitalizations (17), decreased mobility (18–20), and increased risk of complications (21). Hip fractures in the elderly are related to impaired mobility, increased morbidity, and mortality (22), whereas patients aged 60 years or younger with hip fractures experience a low mortality rate, reduced pain severity, and satisfactory functional outcomes 1 year after surgery (23).

Intriguingly, for the first time, we found that the relations of physical pre-frailty and frailty with bone fractures were accentuated by a sedentary lifestyle. The risk of total fractures, hip fractures, and other fractures was stronger in participants who reported longer sedentary behavior time than those with shorter sedentary time. Additionally, we found that participants with frailty and sedentary behavior time >6 h/d had the highest risk of incident bone fractures. Prolonged sedentary behavior time may lead to physical inactivity, which reduces bone cell renewal and repair in the body (24). In addition, prolonged sedentary behavior time causes the bones not to withstand the stimulation of gravity for a long time, leading to bone mass reduction, which may increase the risk of bone fractures (25). Our findings suggest that pre-frail and frail people should avoid long sedentary behavior time to reduce the risk of fractures.

In addition, we observed that the associations of physical frailty with the risk of total fractures were stronger in participants who were older than 60 years, men, with low household income, and current smoker. As people age, their physical function declines, including a reduction in muscle mass and bone density (26). Compared with women, men are more likely to engage in high-risk activities such as extreme sports, high-intensity weightlifting, or activities with a high risk of falls and collisions, which increase the risk of fractures (27). Socioeconomic status is strongly linked to health behaviors that can influence fracture risk. Factors such as smoking, high alcohol consumption, and physical inactivity can increase fracture risk by negatively affecting BMD and/or increasing the risk of falls. These unhealthy behaviors are more prevalent in lower socioeconomic groups compared to higher ones (28,29). Additionally, people with low economic income may have an unbalanced diet (30), resulting in a lack of essential nutrients including calcium and vitamin D, thereby increasing the risk of fractures. Smoking affects the function of the metabolic and circulatory systems, preventing the body from effectively absorbing and utilizing essential nutrients such as calcium, and the chemicals in tobacco damage the cells and matrix in the bones, resulting in decreased bone density, which increases the risk of fractures (31). Therefore, being older than 60 years, male, having a lower income, and smoking may magnify the associations between physical frailty and risk of bone fractures. Several of these factors are potentially modifiable. Smoking is a behavior closely related to daily habits and personal choices and can be modified through lifestyle changes. Household income, although part of socioeconomic status, indirectly influences lifestyle and health behaviors, such as diet quality and health habits. Individuals with lower income are more likely to have an unbalanced diet, which can be mitigated to some extent by improving dietary practices and nutritional supplementation. Addressing these modifiable lifestyle factors may help reduce the risk of fractures associated with physical frailty.

Our study underscores the critical role of physical frailty status in elevating the risk of bone fractures in multiple locations, which suggests the need for heightened vigilance regarding the risk of bone fractures, even during the pre-frailty stage. Furthermore, our research emphasizes the significant interaction between frailty and sedentary behavior time in relation to bone fracture risk. As such, individuals with physical pre-frailty and frailty should strive to decrease their sedentary behavior time to mitigate the risk of bone fractures. Public health messages and clinical advice should prioritize efforts to improve frail status and reduce sedentary behavior time. Additionally, the relationship between low physical activity and frailty is indeed cyclical and interdependent. Low physical activity can lead to physical deconditioning, which contributes to the development of frailty by reducing muscle strength, endurance, and overall physical function. This, in turn, can make it more difficult for individuals to engage in physical activities, thereby perpetuating a cycle of declining physical activity and increasing frailty. In our study, we analyzed the association of each component for physical frailty including physical activity with risk of bone fractures. We observed that physical activity was related to increased risk of incident fractures, which highlights the importance of physical activity in frailty components.

The major strengths of the present study include the large sample size, the consistent results in several sensitivity and subgroup analyses. This study also has some limitations. First, the assessment of the frailty index was indeed based on self-reported answers, which introduces the possibility of misclassification and recall bias. Misclassification could potentially affect our results by causing some participants to be incorrectly classified in terms of their frailty status. This could lead to an attenuation or inflation of the observed associations between frailty status and fracture risks. Second, although we have adjusted for potential confounders, we cannot completely rule out residual confounders. Third, as our study is an observational study, a causal relationship between physical frailty and bone fractures is unable to be determined. Fourth, the UK Biobank is not representative of the general population because of the voluntary participation. Therefore, further studies are needed to confirm our findings.

## Conclusion

Results from this study indicate that both physical pre-frailty and frailty are associated with higher risks of bone fractures at multiple sites than physical nonfrailty. Prolonged sedentary behavior time may strengthen the associations of physical pre-frailty and frailty with bone fractures.

## Supplementary Material

glae186_suppl_Supplementary_Tables_S1-S7_Figures_S1-S5

## Data Availability

This study has been conducted using the UK Biobank Resource, approved project number 29256. The UK Biobank will make the source data available to all bona fide researchers for all types of health-related research that is in the public interest, without preferential or exclusive access for any persons. All researchers will be subject to the same application process and approval criteria as specified by UK Biobank. For more details on the access procedure, see the UK Biobank website: http://www.ukbiobank.ac.uk/register-apply.
