# Supplementary material for: Frailty Status, Sedentary Behaviors, and Risk of Incident Bone Fractures
Source: J Gerontol A Biol Sci Med Sci. 2024 Aug 1;79(9):glae186. doi: 10.1093/gerona/glae186 (PMC11333823; doi:10.1093/gerona/glae186)
Supplement: glae186_suppl_Supplementary_Tables_S1-S7_Figures_S1-S5 [file glae186_suppl_supplementary_tables_s1-s7_figures_s1-s5.docx]

**
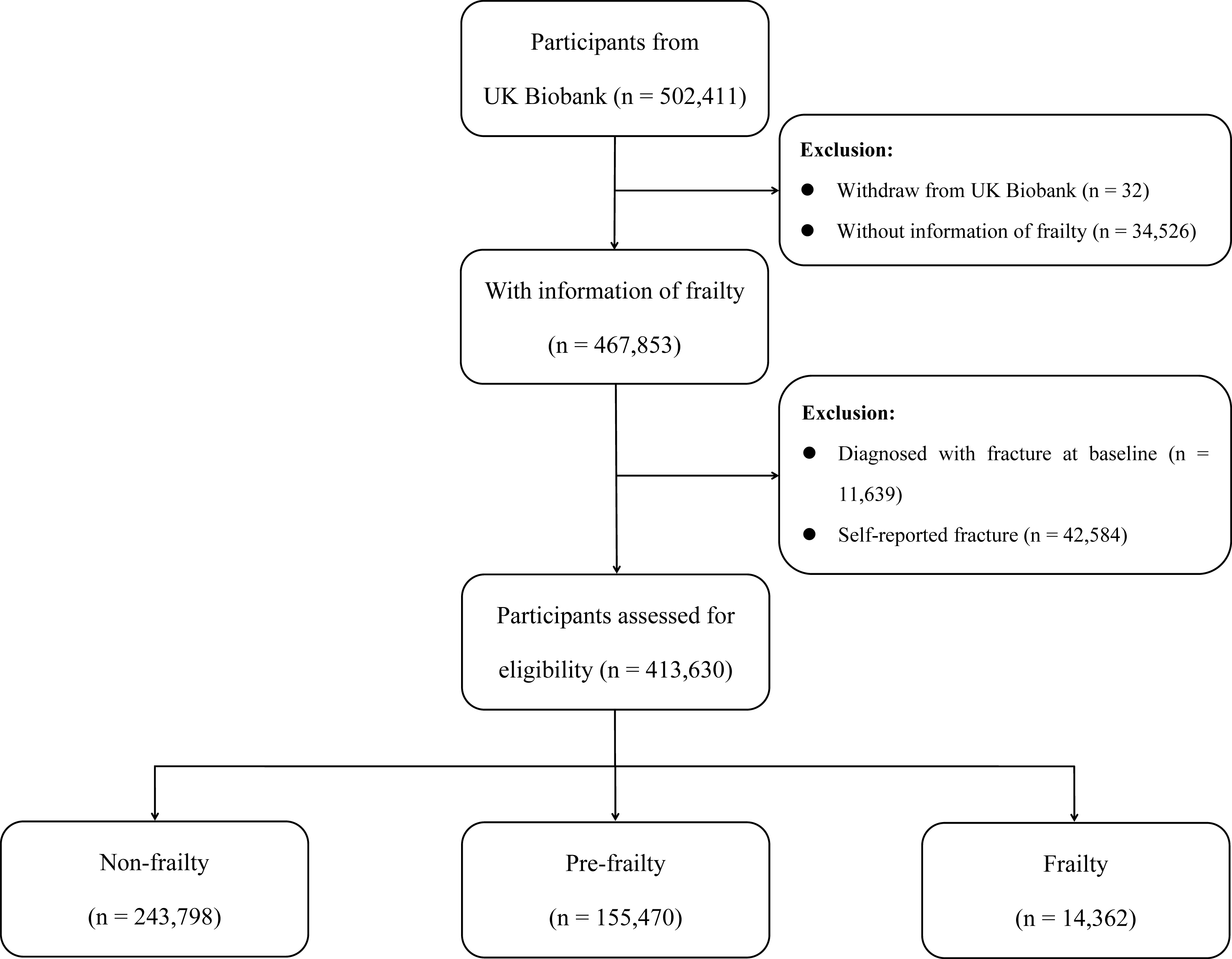
**

**Supplement Figure 1.** Flowchart of participant selection.


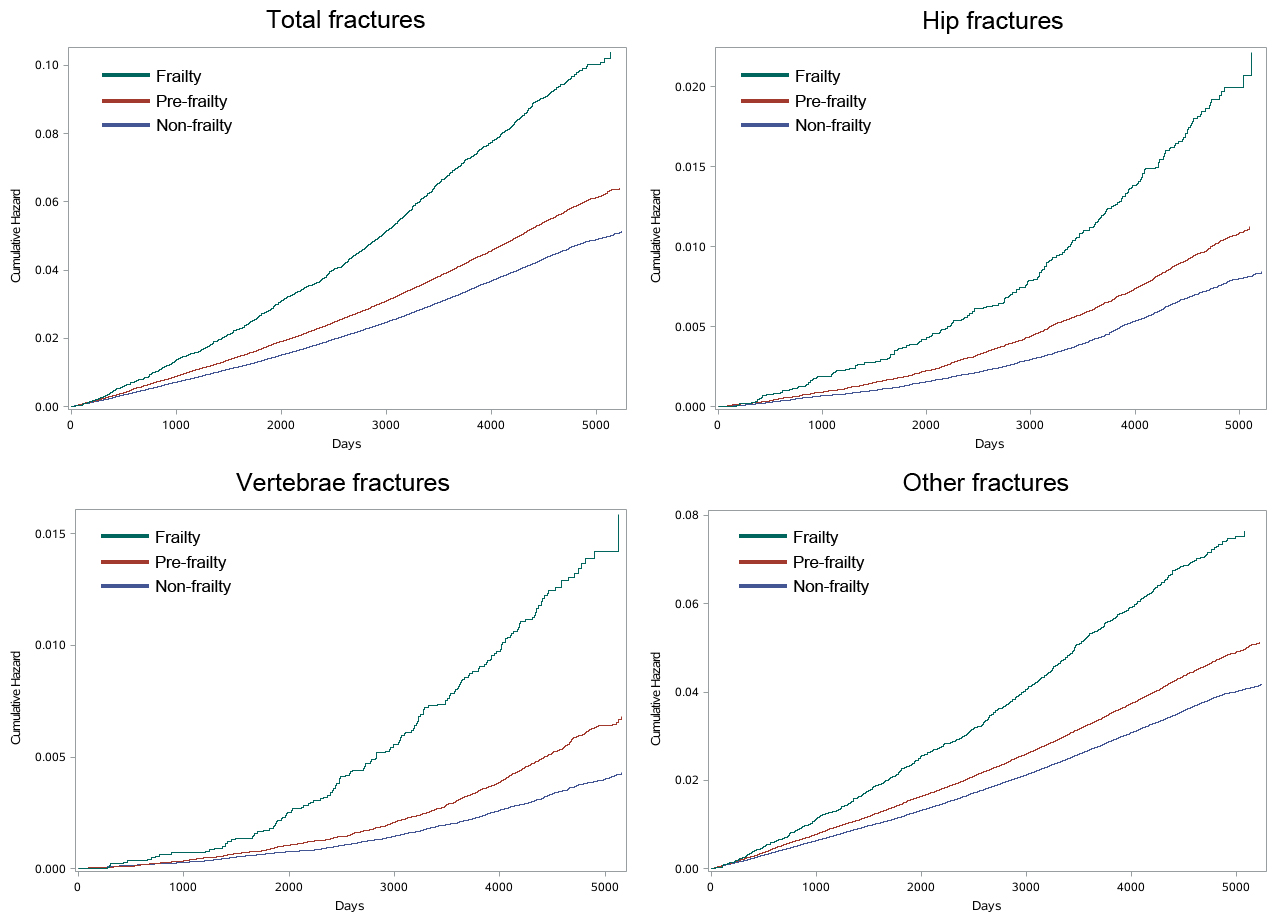


**Supplement Figure 2.** Cumulative hazard curves for the probability of bone fractures in participants with different physical frailty status.

**
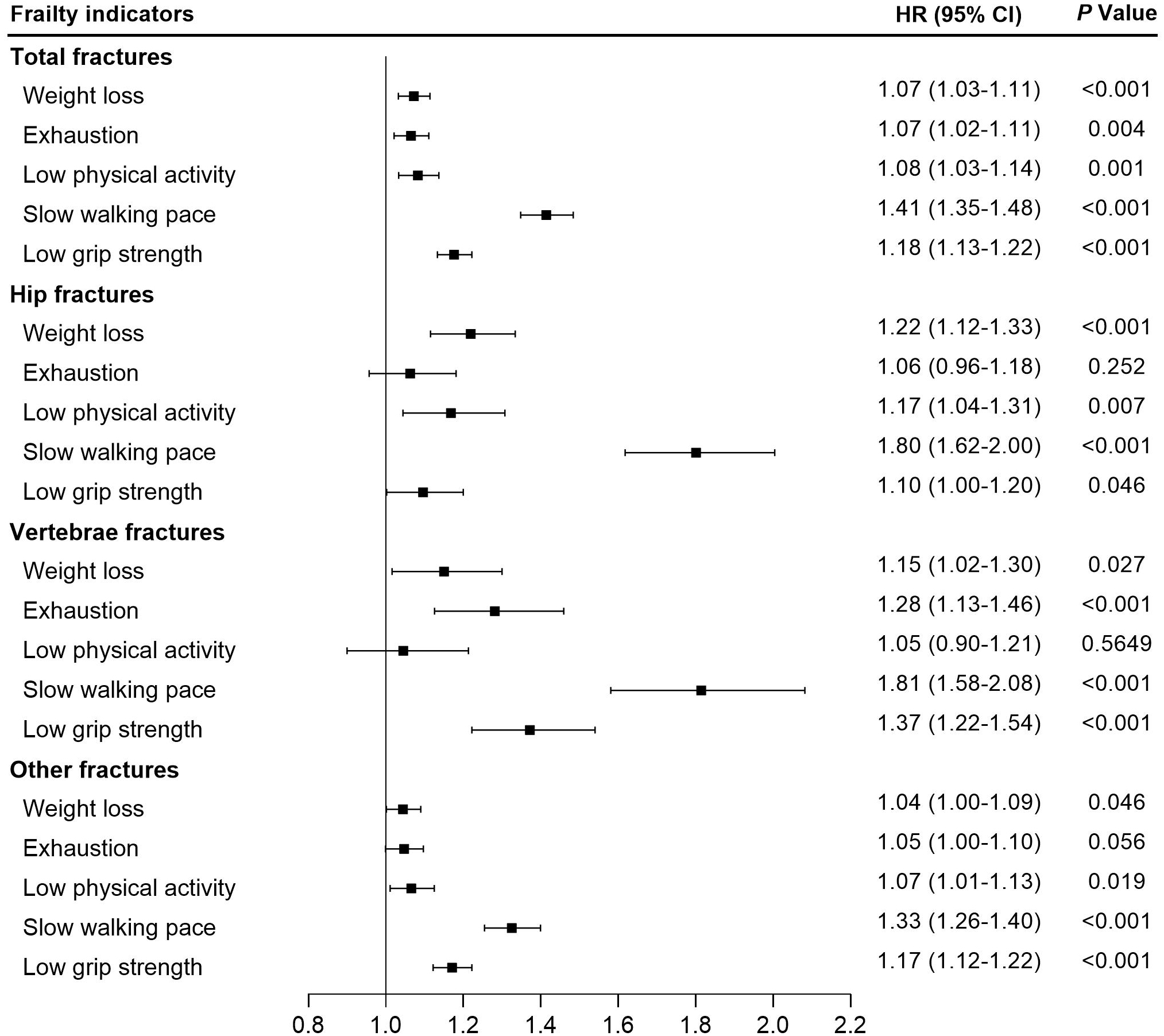
**

**Supplement Figure 3.** Association of individual components of physical frailty with risk of bone fractures via model 3 adjusted for age (years), sex (male or female), ethnic background (white or others), Townsend Deprivation Index (continuous), household income (<£18,000, £18,000-£30,999, £31,000-£51,999, £52,000-£100,000, or >£100,000), body mass index (continuous), standing height (continuous), smoking status (never, previous or current smoking), alcohol intake (<1, 1-2, >2 times/week), healthy diet score (<3 or ≥3), sedentary behavior time (continuous), heel bone mineral density T-score (continuous), falls history (with or without), vitamin D supplementation (yes or no), calcium supplementation (yes or no), serum vitamin D (continuous) and serum calcium (continuous). Weight loss, exhaustion, physical activity, walking pace and grip strength were mutually adjusted for each other.

**
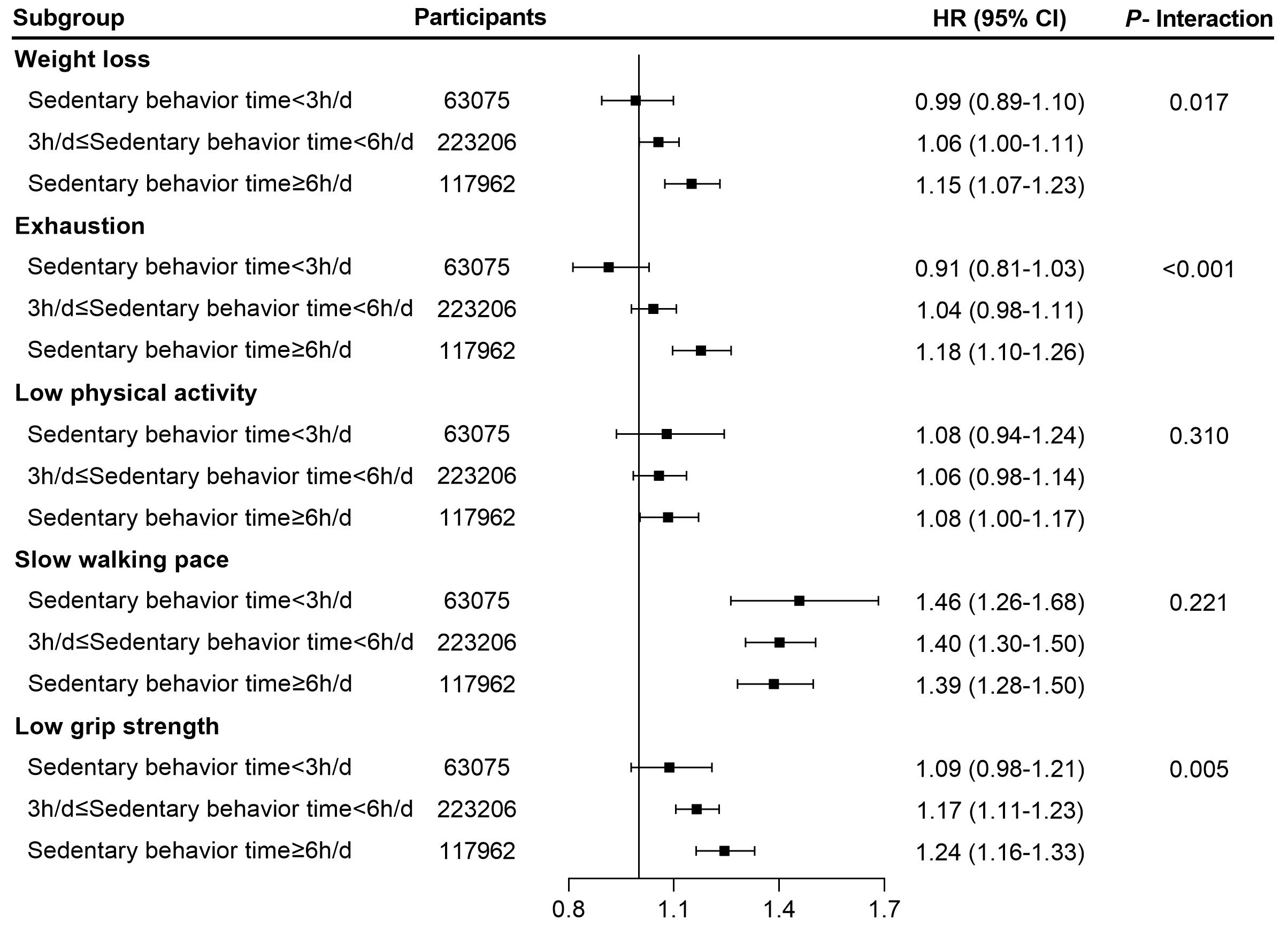
Supplement Figure 4.** Association of physical frailty indicators with risk of total fractures by sedentary behavior time via model 3 adjusted for age (years), sex (male or female), ethnic background (white or others), Townsend Deprivation Index (continuous), household income (<£18,000, £18,000-£30,999, £31,000-£51,999, £52,000-£100,000, or >£100,000), body mass index (continuous), standing height (continuous), smoking status (never, previous or current smoking), alcohol intake (<1, 1-2, >2 times/week), healthy diet score (<3 or ≥3), sedentary behavior time (continuous), heel bone mineral density T-score (continuous), falls history (with or without), vitamin D supplementation (yes or no), calcium supplementation (yes or no), serum vitamin D (continuous) and serum calcium (continuous). Weight loss, exhaustion, physical activity, walking pace and grip strength were mutually adjusted for each other.


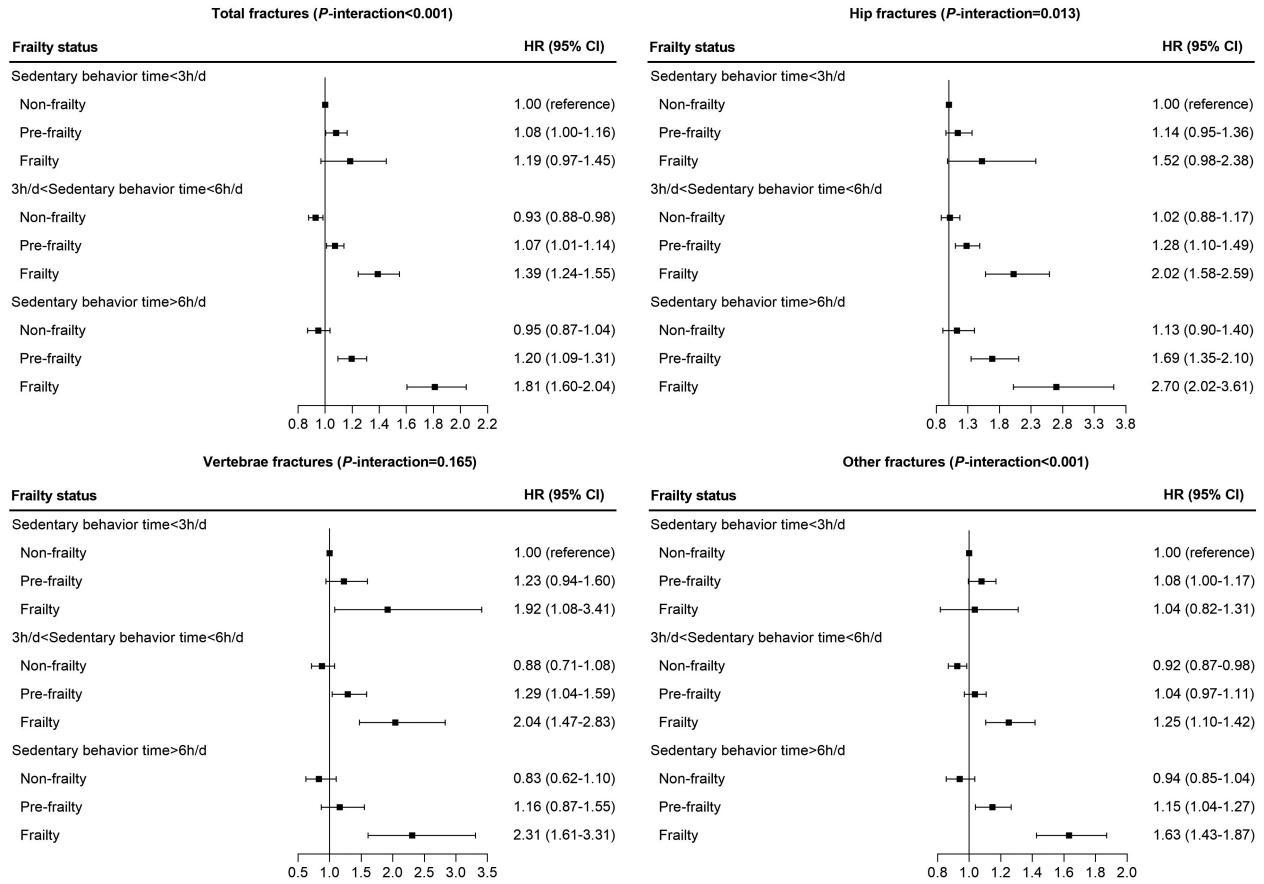


**Supplement Figure 5.** Joint association of frailty status with sedentary behavior time in relation to risk of bone fractures via model 3 adjusted for age (years), sex (male or female), ethnic background (white or others), Townsend Deprivation Index (continuous), household income (<£18,000, £18,000-£30,999, £31,000-£51,999, £52,000-£100,000, or >£100,000), body mass index (continuous), standing height (continuous), smoking status (never, previous or current smoking), alcohol intake (<1, 1-2, >2 times/week), healthy diet score (<3 or ≥3), sedentary behavior time (continuous), heel bone mineral density T-score (continuous), falls history (with or without), vitamin D supplementation (yes or no), calcium supplementation (yes or no), serum vitamin D (continuous) and serum calcium (continuous). Weight loss, exhaustion, physical activity, walking pace and grip strength were mutually adjusted for each other.

**Supplement Table 1.** Physical frailty index criteria.

| **Physical frailty indicators** | **UK Biobank field ID** | **ACE touchscreen question** | **Physical frailty index** |
| --- | --- | --- | --- |
| Weight loss | 2306 | Compared with one year ago, has your weight changed? | - Lost weight=1 - Other=0 |
| Exhaustion | 2080 | Over the past two weeks, how often have you felt tired or had little energy? | - More than half the days or nearly every day=1 - Other=0 |
| Physical activity | 6164 | In the last 4 weeks did you spend any time doing the following? (You can select more than one answer) | - No activity=1 - Medium or heavy activity=0 |
|  | 1011 | How many times in the last 4 weeks did you do light do-it-yourself (DIY) (e.g. pruning, watering the lawn)? | - Light activity with a frequency of once per week or less=1 - Light activity more than once per week=0 |
| Walking pace | 924 | How would you describe your usual walking pace? | - Slow=1 - Other=0 |
| Grip strength | 31 | Sex of participant. | - Maximal grip strength of left and right hands. - Sex and body-mass index adjusted. |
|  | 46 | Left grip strength. |  |
|  | 47 | Right grip strength. |  |
|  | 21001 | BMI value here is constructed from height and weight measured during the initial Assessment Centre visit. |  |

**Supplement Table 2.** ICD 10 codes for fractures definition (UK Biobank field ID: 41270).

| **Outcome** | **Codes** |
| --- | --- |
| Total fractures | M484, M4840, M4841, M4842, M4843, M4844, M4845, M4846, M4847, M4848, M4849, M485, M4850, M4851, M4852, M4853, M4854, M4855, M4856, M4857, M4858, M4859, M800, M8000, M8001, M8002, M8003, M8004, M8005, M8006, M8007, M8008, M8009, M808, M8080, M8081, M8082, M8083, M8084, M8085, M8086, M8087, M8088, M8089, M8090, M8091, M8092, M8093, M8095, M8096, M8097, M8098, M8099, M843, M8430, M8431, M8432, M8433, M8434, M8435, M8436, M8437, M8438, M8439, M844, M8440, M8441, M8442, M8443, M8444, M8445, M8446, M8447, M8448, M8449, S12, S120, S1200, S1201, S121, S1210, S1211, S122, S1220, S1221, S127, S1270, S1271, S128, S1280, S1281, S129, S1290, S1291, S22, S220, S2200, S2201, S221, S2210, S2211, S222, S2220, S2221, S223, S2230, S2231, S224, S2240, S2241, S225, S2250, S2251, S228, S2280, S2281, S229, S2290, S2291, S32, S320, S3200, S3201, S321, S3210, S3211, S322, S3220, S3221, S323, S3230, S3231, S324, S3240, S3241, S325, S3250, S3251, S327, S3270, S3271, S328, S3280, S3281, S42, S420, S4200, S4201, S421, S4210, S4211, S422, S4220, S4221, S423, S4230, S4231, S424, S4240, S4241, S427, S4270, S4271, S428, S4280, S4281, S429, S4290, S4291, S52, S520, S5200, S5201, S521, S5210, S5211, S522, S5220, S5221, S523, S5230, S5231, S524, S5240, S5241, S525, S5250, S5251, S526, S5260, S5261, S527, S5270, S5271, S528, S5280, S5281, S529, S5290, S5291, S72, S720, S7200, S7201, S721, S7210, S7211, S722, S7220, S7221, S723, S7230, S7231, S724, S7240, S7241, S727, S7270, S7271, S728, S7280, S7281, S729, S7290, S7291, S82, S820, S8200, S8201, S821, S8210, S8211, S822, S8220, S8221, S823, S8230, S8231, S824, S8240, S8241, S825, S8250, S8251, S826, S8260, S8261, S827, S8270, S8271, S828, S8280, S8281, S8286, S829, S8290, S8291, T02, T020, T0200, T0201, T021, T0210, T0211, T022, T0220, T0221, T023, T0230, T0231, T024, T0240, T0241, T025, T0250, T0251, T026, T0260, T0261, T027, T0270, T0271, T028, T0280, T0281, T029, T0290, T0291, T08, T08X0, T08X1, T10, T10X0, T10X1, T12, T12X0, T12X1. |
| Hip fractures | S72, S720, S7200, S7201, S721, S7210, S7211, S722, S7220, S7221. |
| Vertebrae fractures | M484, M4840, M4841, M4842, M4843, M4844, M4845, M4846, M4847, M4848, M4849, M485, M4850, M4851, M4852, M4853, M4854, M4855, M4856, M4857, M4858, M4859, S32, S320, S3200, S3201, S327, S3270, S3271. |
| Other fractures | M800, M8000, M8001, M8002, M8003, M8004, M8005, M8006, M8007, M8008, M8009, M808, M8080, M8081, M8082, M8083, M8084, M8085, M8086, M8087, M8088, M8089, M8090, M8091, M8092, M8093, M8095, M8096, M8097, M8098, M8099, M843, M8430, M8431, M8432, M8433, M8434, M8435, M8436, M8437, M8438, M8439, M844, M8440, M8441, M8442, M8443, M8444, M8445, M8446, M8447, M8448, M8449, S12, S120, S1200, S1201, S121, S1210, S1211, S122, S1220, S1221, S127, S1270, S1271, S128, S1280, S1281, S129, S1290, S1291, S22, S220, S2200, S2201, S221, S2210, S2211, S222, S2220, S2221, S223, S2230, S2231, S224, S2240, S2241, S225, S2250, S2251, S228, S2280, S2281, S229, S2290, S2291, S321, S3210, S3211, S322, S3220, S3221, S323, S3230, S3231, S324, S3240, S3241, S325, S3250, S3251, S328, S3280, S3281, S42, S420, S4200, S4201, S421, S4210, S4211, S422, S4220, S4221, S423, S4230, S4231, S424, S4240, S4241, S427, S4270, S4271, S428, S4280, S4281, S429, S4290, S4291, S52, S520, S5200, S5201, S521, S5210, S5211, S522, S5220, S5221, S523, S5230, S5231, S524, S5240, S5241, S525, S5250, S5251, S526, S5260, S5261, S527, S5270, S5271, S528, S5280, S5281, S529, S5290, S5291, S723, S7230, S7231, S724, S7240, S7241, S727, S7270, S7271, S728, S7280, S7281, S729, S7290, S7291, S82, S820, S8200, S8201, S821, S8210, S8211, S822, S8220, S8221, S823, S8230, S8231, S824, S8240, S8241, S825, S8250, S8251, S826, S8260, S8261, S827, S8270, S8271, S828, S8280, S8281, S8286, S829, S8290, S8291, T02, T020, T0200, T0201, T021, T0210, T0211, T022, T0220, T0221, T023, T0230, T0231, T024, T0240, T0241, T025, T0250, T0251, T026, T0260, T0261, T027, T0270, T0271, T028, T0280, T0281, T029, T0290, T0291, T08, T08X0, T08X1, T10, T10X0, T10X1, T12, T12X0, T12X1. |

| **Category** | **UK Biobank field ID** | **Codes or ACE touchscreen question** |
| --- | --- | --- |
| ICD 9 | 41271 | 7338, 73381, 73382, 73383, 73384, 73385, 73386, 73387, 73388, 73389; 800, 8000, 8001, 8002, 8003, 801, 8010, 8011, 802, 8020, 8022, 8023, 8024, 8026, 8028, 803, 8030, 8031; 805, 8050, 8052, 8054, 8056, 8058; 806, 8064, 807, 8070, 8072, 8074, 808, 8080, 8082, 8084, 8088, 8089, 809, 8090, 8091;810, 8100, 811, 8110, 812, 8120, 8121, 8122, 8123, 8124, 8125, 813, 8130, 8131, 8132, 8134, 8135, 814,8140, 8141, 815, 8150, 8151, 816, 8160, 8161, 817, 8170; 820, 8200, 8202, 8208, 8210, 8211, 8212; 822, 8220, 8221; 823, 8230, 8231, 8232, 8233, 8240, 8241, 8242, 8244, 8245, 8246, 8247, 8248, 8249, 825, 8250, 8252, 8253, 826, 8260, 8261, 828, 8280, 829, 8290; 905, 9050, 9052, 9053, 9054. |
| Self-report | 20002 | 1626, 1627, 1628, 1629,1630, 1631, 1632, 1633, 1634, 1635, 1636, 1637, 1638, 1639, 1640, 1644, 1645, 1646, 1647, 1648, 1649,1650, 1651, 1652, 1653, 1654, 1655, 1656. |
| Self-report | 2463 | Have you fractured/broken any bones in the last 5 years? |

**Supplement Table 3.** ICD 9 and self-report codes for fractures definition. ICD 9 and self-report diagnoses were used only to ascertain the presence of bone fractures at baseline for exclusion from incident analyses.

**Supplement Table 4.** The numbers and percentages of participants with missing covariates.

| **Variable** | **N** | **%** |
| --- | --- | --- |
| Serum calcium | 56041 | 13.55 |
| Household income | 56035 | 13.55 |
| Heel BMD T-score | 43931 | 10.62 |
| Serum vitamin D | 40242 | 9.73 |
| Healthy diet score | 13949 | 3.37 |
| Sedentary behavior time | 9387 | 2.27 |
| Vitamin D supplementation | 1491 | 0.36 |
| Ethnic background | 1293 | 0.31 |
| Smoking status | 1281 | 0.31 |
| Calcium supplementation | 690 | 0.17 |
| Fall history | 685 | 0.17 |
| Townsend deprivation index | 513 | 0.12 |
| Alcohol intake | 235 | 0.06 |

**Supplement Table 5.** Hazard ratios and 95% confidence intervals obtained from model 3^*^ for association of physical frailty status with outcome of fractures after removing the participants with fractures within 2 years.

| **Outcomes** | **Physical frailty status** | | | ***P-*trend** |
| --- | --- | --- | --- | --- |
|  | **Non-frailty** | **Pre-frailty** | **Frailty** |  |
| Total fractures | 1 (references) | 1.17 (1.13-1.21) | 1.68 (1.57-1.80) | <0.001 |
| Hip fractures | 1 (references) | 1.30 (1.21-1.40) | 2.05 (1.76-2.39) | <0.001 |
| Vertebrae fractures | 1 (references) | 1.43 (1.29-1.59) | 2.60 (2.15-3.14) | <0.001 |
| Other fractures | 1 (references) | 1.14 (1.10-1.18) | 1.53 (1.42-1.66) | <0.001 |

**^*^Model 3:** adjusted for age (years), sex (male or female), ethnic background (white or others), Townsend Deprivation Index (continuous), household income (<£18,000, £18,000-£30,999, £31,000-£51,999, £52,000-£100,000, or >£100,000), body mass index (continuous), standing height (continuous), smoking status (never, previous or current smoking), alcohol intake (<1, 1-2, >2 times/week), healthy diet score (<3 or ≥3), sedentary behavior time (continuous), heel bone mineral density T-score (continuous), falls history (with or without), vitamin D supplementation (yes or no), calcium supplementation (yes or no), serum vitamin D (continuous) and serum calcium (continuous).

**Supplement Table 6.** Hazard ratios and 95% confidence intervals obtained from model 3^*^ for association of physical frailty status with outcome of fractures with all missing covariate data deleted.

| **Outcomes** | **Physical frailty status** | | | ***P*-trend** |
| --- | --- | --- | --- | --- |
|  | **Non-frailty** | **Pre-frailty** | **Frailty** |  |
| Total fractures | 1 (references) | 1.14 (1.10-1.19) | 1.67 (1.52-1.83) | <0.001 |
| Hip fractures | 1 (references) | 1.29 (1.17-1.43) | 2.20 (1.78-2.72) | <0.001 |
| Vertebrae fractures | 1 (references) | 1.36 (1.19-1.56) | 2.62 (2.02-3.40) | <0.001 |
| Other fractures | 1 (references) | 1.11 (1.06-1.16) | 1.48 (1.33-1.64) | <0.001 |

**^*^Model 3:** adjusted for age (years), sex (male or female), ethnic background (white or others), Townsend Deprivation Index (continuous), household income (<£18,000, £18,000-£30,999, £31,000-£51,999, £52,000-£100,000, or >£100,000), body mass index (continuous), standing height (continuous), smoking status (never, previous or current smoking), alcohol intake (<1, 1-2, >2 times/week), healthy diet score (<3 or ≥3), sedentary behavior time (continuous), heel bone mineral density T-score (continuous), falls history (with or without), vitamin D supplementation (yes or no), calcium supplementation (yes or no), serum vitamin D (continuous) and serum calcium (continuous).

**Supplement Table 7.** Hazard ratios and 95% confidence intervals obtained from model 3^*^ for association of physical frailty status with outcome of fractures with all missing covariate data imputed using multiple imputation.

| **Outcomes** | **Physical frailty status** | | | ***P*-trend** |
| --- | --- | --- | --- | --- |
|  | **Non-frailty** | **Pre-frailty** | **Frailty** |  |
| Total fractures | 1 (references) | 1.18 (1.14-1.21) | 1.66 (1.56-1.77) | <0.001 |
| Hip fractures | 1 (references) | 1.31 (1.22-1.41) | 2.10 (1.81-2.43) | <0.001 |
| Vertebrae fractures | 1 (references) | 1.43 (1.29-1.58) | 2.60 (2.17-3.12) | <0.001 |
| Other fractures | 1 (references) | 1.14 (1.11-1.18) | 1.51 (1.40-1.62) | <0.001 |

**^*^Model 3:** adjusted for age (years), sex (male or female), ethnic background (white or others), Townsend Deprivation Index (continuous), household income (<£18,000, £18,000-£30,999, £31,000-£51,999, £52,000-£100,000, or >£100,000), body mass index (continuous), standing height (continuous), smoking status (never, previous or current smoking), alcohol intake (<1, 1-2, >2 times/week), healthy diet score (<3 or ≥3), sedentary behavior time (continuous), heel bone mineral density T-score (continuous), falls history (with or without), vitamin D supplementation (yes or no), calcium supplementation (yes or no), serum vitamin D (continuous) and serum calcium (continuous).
